# Supplementary material for: Real World Cost-Effectiveness Analysis of Population Screening for BRCA Variants among Ashkenazi Jews Compared with Family History-Based Strategies
Source: Cancers (Basel). 2022 Dec 12;14(24):6113. doi: 10.3390/cancers14246113 (PMC9776581; doi:10.3390/cancers14246113)
Supplement: Supplementary file 1 [file cancers-14-06113-s001.zip › cancers-1952223-supplementary.pdf]

Supplements

Table S1. Estimated QALYs.

Health and carrier status	QALY
Unaffected woman	26.74
Noncarrier affected with BC	26.48
Carrier affected with BC	25.73
Noncarrier affected with OC	23.6
Carrier affected with OC	22.1

BC – Breast cancer. OC- Ovarian cancer.

Table S2. Model outcomes for costs, discounted quality-adjusted life-years (QALYs) and probability.

A	PS	Probability	Cost (K, NIS)	QALYs
1	test, carrier, RRSO, RRM, no OC/BC	0.0811%	131.72	26.74
2	test, carrier, RRSO, RRM, OC	0.0011%	360.8	22.1
3	test, carrier, RRSO, RRM, BC	0.0018%	438.82	25.73
4	test, carrier, RRSO, no RRM, no OC/BC	1.0156%	186.21	26.74
5	test, carrier, RRSO, no RRM, OC	0.0165%	415.28	22.1
6	test, carrier, RRSO, no RRM, BC	0.2827%	493.3	25.73
7	test, carrier, no RRSO, RRM, no OC/BC	0.0107%	115.61	26.74
8	test, carrier, no RRSO, RRM, OC	0.0052%	344.69	22.1
9	test, carrier, no RRSO, RRM, BC	0.0007%	422.71	25.73
10	test, carrier, no RRSO, no RRM, no OC/BC	0.0668%	170.1	26.74
11	test, carrier, no RRSO, no RRM, OC	0.0813%	399.17	22.1
12	test, carrier, no RRSO, no RRM, BC	0.1117%	477.19	25.73
13	test, non-carrier, no OC/BC	55.8529%	48.29	26.74
14	test, non-carrier, OC	0.9799%	291.01	23.6
15	test, non-carrier, BC	8.4923%	355.38	26.48
16	not test, carrier, no OC/BC	0.2120%	48.21	26.74
17	not test, carrier, OC	0.2582%	290.93	22.1
18	not test, carrier, BC	0.3548%	355.3	25.73
19	not test, non-carrier, no OC/BC	27.5096%	48.21	26.74
20	not test, non-carrier, OC	0.4826%	290.93	23.6
21	not test, non-carrier, BC	4.1827%	355.3	26.48
**B**	**CT**	**Probability**	**Cost (K,NIS)**	**QALYs**
22	get tested, carrier, RRSO, RRM, no OC/BC	0.0137%	132.28	26.74
23	get tested, carrier, RRSO, RRM, OC	0.0002%	361.35	22.1
24	get tested, carrier, RRSO, RRM, BC	0.0003%	439.37	25.73
25	get tested, carrier, RRSO, no RRM, no OC/BC	0.1719%	186.76	26.74
26	get tested, carrier, RRSO, no RRM, OC	0.0028%	415.83	22.1
79	get tested, carrier, RRSO, no RRM, BC	0.0479%	493.86	25.73
28	get tested, carrier, no RRSO, RRM, no OC/BC	0.0018%	116.17	26.74
29	get tested, carrier, no RRSO, RRM, OC	0.0009%	345.24	22.1
30	get tested, carrier, no RRSO, RRM, BC	0.0001%	423.26	25.73
31	get tested, carrier, no RRSO, no RRM, no OC/BC	0.0113%	170.65	26.74
32	get tested, carrier, no RRSO, no RRM, OC	0.0138%	399.73	22.1
33	get tested, carrier, no RRSO, no RRM, BC	0.0189%	477.75	25.73
34	get tested, non-carrier, no OC/BC	0.3390%	49.48	26.74
35	get tested, non-carrier, OC	0.0059%	292.2	23.6
36	get tested, non-carrier, BC	0.0515%	356.57	26.48
37	not tested, carrier, no OC/BC	0.6381%	48.21	26.74
38	not tested, carrier, OC	0.7772%	290.93	22.1
39	not tested, carrier, BC	1.0677%	355.3	25.73
40	not tested, non-carrier, no OC/BC	82.7956%	48.21	26.74
41	not tested, non-carrier, OC	1.4526%	290.93	23.6
42	not tested, non-carrier, BC	12.5888%	355.3	26.48
**C**	**IFH**	**Probability**	**Cost (K,NIS)**	**QALYs**
43	get tested, carrier, RRSO, RRM, no OC/BC	0.0208%	132.28	26.74
44	get tested, carrier, RRSO, RRM, OC	0.0003%	351.35	22.1
45	get tested, carrier, RRSO, RRM, BC	0.0005%	439.37	25.73

46	get tested, carrier, RRSO, no RRM, no OC/BC	0.2601%	186.76	26.74
47	get tested, carrier, RRSO, no RRM, OC	0.0042%	415.83	22.1
48	get tested, carrier, RRSO, no RRM, BC	0.0724%	493.86	25.73
49	get tested, carrier, no RRSO, RRM, no OC/BC	0.0027%	116.17	26.74
50	get tested, carrier, no RRSO, RRM, OC	0.0013%	345.24	22.1
51	get tested, carrier, no RRSO, RRM, BC	0.0002%	423.26	25.73
52	get tested, carrier, no RRSO, no RRM, no OC/BC	0.0171%	170.65	26.74
53	get tested, carrier, no RRSO, no RRM, OC	0.0208%	399.73	22.1
54	get tested, carrier, no RRSO, no RRM, BC	0.0286%	477.75	25.73
55	get tested, non-carrier, no OC/BC	9.0382%	49.48	26.74
56	get tested, non-carrier, OC	0.1586%	292.2	23.6
57	get tested, non-carrier, BC	1.3742%	356.57	26.48
58	not tested, carrier, no OC/BC	0.3888%	48.21	26.74
59	not tested, carrier, OC	0.4736%	290.93	22.1
60	not tested, carrier, BC	0.6506%	355.3	25.73
61	not tested, non-carrier, no OC/BC	74.8014%	48.21	26.74
62	not tested, non-carrier, OC	1.3123%	290.93	23.6
63	not tested, non-carrier, BC	11.3733%	355.3	26.48

PS- population screening, CT –cascade testing, IFH-International Family History strategy/ H – Healthy (unaffected with cancer). BC – Breast Cancer. OC –Ovarian Cancer. RRM – risk-reduction mastectomy. RRSO – risk reduction salpingo-oophorectomy.

Table S3. The effect of RRSO on BC risk – sensitivity analysis.

BC RRSO risk reduction	Strategy	Cost (US$)	Incremental Cost (US$)	Effectiveness	Incremental Effectiveness	ICER (US$)
	PS	27016	0	26.406		
0	CT	26881	-134	26.386	-0.020	6750
	IFH	26565	-450	26.401	-0.005	95594
	PS	26967	0	26.406		
0.1	CT	26873	-94	26.386	-0.020	4649
	IFH	26553	-414	26.401	-0.005	82507
	PS	26919	0	26.407		
0.2	CT	26865	-54	26.386	-0.021	2619
	IFH	26540	-378	26.401	-0.005	70947
	PS	26870	0	26.407		
0.3	CT	26857	-14	26.386	-0.021	657
	IFH	26528	-342	26.401	-0.006	60662
	PS	26822	0	26.407		
0.4	CT	26849	26	26.386	-0.021	-1242
	IFH	26516	-333	26.402	0.015	-21720
	PS	26774	0	26.408		
0.5	CT	26840	67	26.386	-0.022	-3079
	IFH	26503	-337	26.402	0.015	-21942

BC – Breast Cancer. PS – population screening. CT –cascade testing. IFH- international family history. RRSO– risk reduction salpingo-oophorectomy

Table S4. Carrier prevalence in women tested in PS – sensitivity analysis.

PS			PS vs CT			PS vs IFH		
**Carrier rate (PS)**[a]	Cost (USD)	Effectiveness (QALY)	Incremental cost (CT-26990 USD)	Incremental effectiveness (CT-26.39 QALY)	ICER/QALY (USD)	Incremental cost (IFH-26651 USD)	Incremental effectiveness (IFH-26.402 QALY)	ICER/QALY (USD)
0.015	26594.4	26.41	-396.08	0.024	-16503.3	-57.42	0.008	-6,864.71
0.022	26824.93	26.41	-165.83	0.022	-7537.56	173.11	0.008	20,694.4
0.029	27055.46	26.41	64.71	0.021	3081.232	403.64	0.008	48,253.5

IFH – International family-history based. CT –cascade testing. PS – population screening.
[a] In this analysis, carrier prevalence in the CT, IFH strategies were constant (See Methods).

Table S5. One way sensitivity analysis for genetic testing costs: PS vs. CT strategies.

cost of ps test (US$)	Strategy	Cost (US$)	Incr Cost (US$)	Effectiveness	Incr Effectiveness	ICER
	PS	26770	0	26.408	0.000	0
16	CT	26840	71	26.386	-0.022	-3274
	IFH	26503	-337	26.402	0.015	-21942
	PS	26794	0	26.408	0.000	0
52.8	CT	26840	46	26.386	-0.022	-2134
	IFH	26503	-337	26.402	0.015	-21942
	PS	26819	0	26.408	0.000	0
89.6	CT	26840	21	26.386	-0.022	-994
	IFH	26503	-337	26.402	0.015	-21942
	PS	26844	0	26.408	0.000	0
126.4	CT	26840	-3	26.386	-0.022	146
	IFH	26503	-340	26.402	-0.006	54288
	PS	26868	0	26.408	0.000	0
163.2	CT	26840	-28	26.386	-0.022	1286
	IFH	26503	-365	26.402	-0.006	58222
	PS	26893	0	26.408	0.000	0
200	CT	26840	-52	26.386	-0.022	2426
	IFH	26503	-390	26.402	-0.006	62157

PS – population screening. CT –cascade testing. IFH- international family history. RRSO– risk reduction salpingo-oophorectomy.

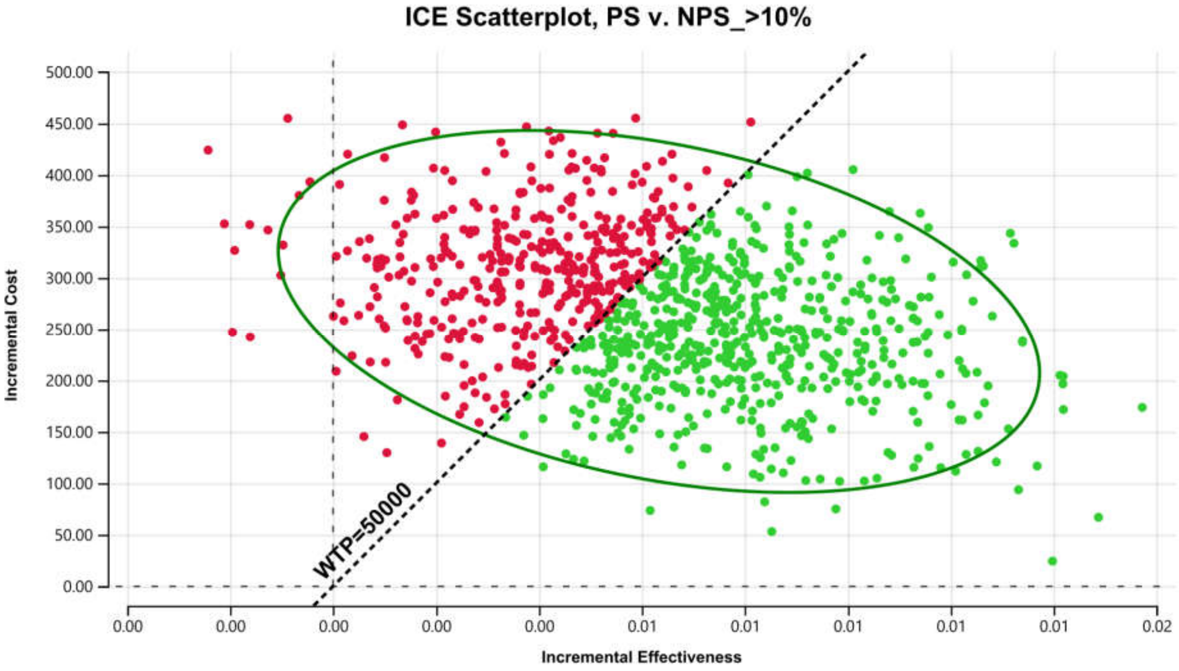

Figure S1. Incremental effectiveness scatterplot, PS vs. non-PS. The green dots represent individuals for whom PS (population screening) was preferable to NPS (non-population screening, the alternative strategy, also known as IFH- international family history based). With a WTP (willingness to pay) of 50,000 USD, most individuals in a cohort of 1000 would prefer PS.
